# Hyperhomocysteinemia and Klotho in kidney-to-brain signaling: a metabolic stress-resilience framework for neurovascular dysfunction

**DOI:** 10.3389/fnins.2026.1989720

**Published:** 2026-10-26

**Authors:** Dongyan Zhou, Yulong Zhao, Yanjian Zheng, Jianying Xu, Bibo Xie, Yuqiu Zhou, Yingpin Zhang

**Affiliations:** Department of Clinical Laboratory, The Fifth People's Hospital of Zhuhai, Zhuhai, China

**Keywords:** blood–brain barrier, homocysteine, hyperhomocysteinemia, kidney-brain axis, neurovascular unit, α-Klotho

## Abstract

Hyperhomocysteinemia (HHcy) and Klotho deficiency are commonly investigated in separate metabolic, renal, vascular, and neurological contexts, yet together they may illuminate how peripheral dysfunction is translated into cerebral vulnerability. This review develops a kidney-to-brain stress resilience framework in which HHcy represents a renal dysfunction-associated component of systemic metabolic and vascular stress, whereas α-Klotho represents a candidate indicator of biological resilience, supported mainly by experimental evidence and observational human studies. Declining renal function favors Hcy accumulation, reduces renal Klotho expression, and generates uremic, inflammatory, mineral, and hemodynamic disturbances that converge on the cerebral microvasculature. Experimental HHcy can additionally repress renal KL through HDAC9-dependent epigenetic regulation. At the brain-facing interface, circulating Hcy directly disrupts cerebral endothelial bioenergetics, redox homeostasis, inflammatory signaling, and tight-junction integrity, providing a plausible route from systemic metabolic disturbance to blood–brain barrier (BBB) and neurovascular-unit (NVU) dysfunction. Klotho-related mechanisms, in contrast, are associated with endothelial, antioxidant, mitochondrial, anti-inflammatory, and anti-senescent resilience, although circulating, renal, and CNS Klotho represent non-equivalent compartments and the causal contribution of circulating Klotho to human neurological disease remains uncertain. Clinical and imaging evidence from chronic kidney disease, cerebral small-vessel disease (CSVD), cognitive impairment, and ischemic stroke supports individual components of the proposed kidney circulation BBB/NVU brain continuum, but not a validated linear HHcy-Klotho molecular axis. We therefore define HHcy-high/Klotho-low status as a candidate metabolic stress-resilience phenotype and propose that the critical test is whether Klotho modifies the neurovascular consequences of a given Hcy burden. Establishing this framework will require cell-specific perturbation, compartment-resolved Klotho measurement, longitudinal neuroimaging, and prospective analyses linking renal dysfunction, BBB/NVU injury, CSVD progression, and cognitive decline.

## 1 Introduction

Brain–body communication is increasingly viewed as a distributed physiological process in which metabolism, immune activity, vascular function, and endocrine signaling outside the central nervous system contribute to neural homeostasis. The kidney is particularly relevant in this setting because loss of renal function alters several of these processes at the same time, and cognitive impairment is common in patients with chronic kidney disease (CKD) (Sammons et al., 2024; Yan et al., 2024). Here, we use the term kidney-brain axis to describe the routes by which these systemic consequences of renal dysfunction become relevant to the brain. The cerebral microvascular endothelium and the blood–brain barrier (BBB) are likely to be important parts of this communication because they are continuously exposed to circulating metabolites and inflammatory or endocrine mediators, while also responding to systemic vascular and hemodynamic changes. Dysfunction at this blood-facing interface can subsequently affect other components of the neurovascular unit (NVU), with consequences for cerebral perfusion, inflammatory activity, and neural homeostasis.

Homocysteine (Hcy) is an important metabolic link in this kidney-centered model. It is produced in the body during methionine and one-carbon metabolism and is not produced specifically by the kidney. Plasma concentrations depend on remethylation and transsulfuration, B-vitamin availability, genetic background, aging, and renal function. Declining kidney function is strongly associated with Hcy accumulation because both Hcy handling and systemic metabolism are altered in CKD. HHcy is therefore more appropriately interpreted as a systemic metabolic abnormality associated with renal dysfunction than as a kidney-derived signal. Clinically, HHcy has been associated with vascular disease and progression from mild cognitive impairment to dementia, while experimental work indicates that elevated Hcy can directly damage cerebral endothelial cells and impair BBB function(Carey et al., 2024; Joshi and Jadavji, 2024; Zuliani et al., 2024). Once present at increased concentrations in blood, Hcy is encountered directly by the luminal surface of brain endothelial cells. This provides a plausible route through which renal and systemic metabolic abnormalities may contribute to cerebral microvascular stress.

Hcy burden, however, describes only one side of this problem. Individuals exposed to a similar degree of metabolic or vascular stress may differ substantially in their ability to maintain endothelial, mitochondrial, redox, inflammatory, and cellular homeostasis. This difference in biological reserve provides the rationale for considering Klotho as a second dimension of the kidney-brain model. Klotho refers specifically to α-Klotho encoded by *KL*. The kidney is the major peripheral site of α-Klotho expression, and soluble Klotho can be detected in the circulation. Its strong renal origin and decline with renal dysfunction make Klotho particularly relevant to a kidney-centered model rather than simply a general anti-aging factor. Klotho biology intersects with oxidative stress, inflammation, endothelial homeostasis, mitochondrial function, cellular senescence, and neurological aging, although the clinical interpretation of soluble Klotho remains limited by assay heterogeneity and inconsistent outcome associations (Edmonston et al., 2024a; Kanbay et al., 2024a; Kanbay et al., 2024b; Czaplicki et al., 2026). Importantly, the stress and resilience dimensions represented by HHcy and Klotho may not be biologically independent. In a defined renal context, experimental HHcy induces HDAC9-dependent epigenetic repression of Klotho in podocytes, providing direct evidence that increased Hcy can suppress a Klotho-related protective mechanism in the kidney (Liu et al., 2023). This finding establishes a specific HHcy-to-Klotho regulatory link in renal cells, but it does not demonstrate that the same interaction occurs in cerebral endothelial cells or other components of the NVU, nor that reduced circulating Klotho mediates the cerebral effects of HHcy.

These findings support a stress-resilience model of kidney-to-brain communication. By this model, we mean a two-dimensional conceptual framework in which neurological vulnerability reflects the balance between the magnitude of systemic injurious stress and the capacity of peripheral and cerebral tissues to maintain homeostasis under stress conditions. HHcy is considered a representative marker of systemic metabolic and vascular stress, whereas Klotho represents a candidate determinant of biological resilience that may influence the ability of tissues to withstand or recover from such stress. Importantly, this framework does not propose a simple linear causal sequence in which HHcy suppresses Klotho and Klotho loss subsequently drives brain injury. Rather, it considers metabolic stress and resilience as partially independent biological dimensions that may converge at the cerebral endothelium, blood–brain barrier (BBB), and neurovascular unit (NVU), where their relative balance may shape neurovascular vulnerability. Therefore, this review aims to evaluate the current evidence supporting each component of the proposed kidney-circulation-BBB/NVU-brain continuum, identify potential points of mechanistic convergence between HHcy-associated stress and Klotho-related resilience, and define the experimental and longitudinal clinical studies required to determine whether Klotho modifies the neurovascular and cognitive consequences of a given Hcy burden (Figure 1).

**Figure 1:**
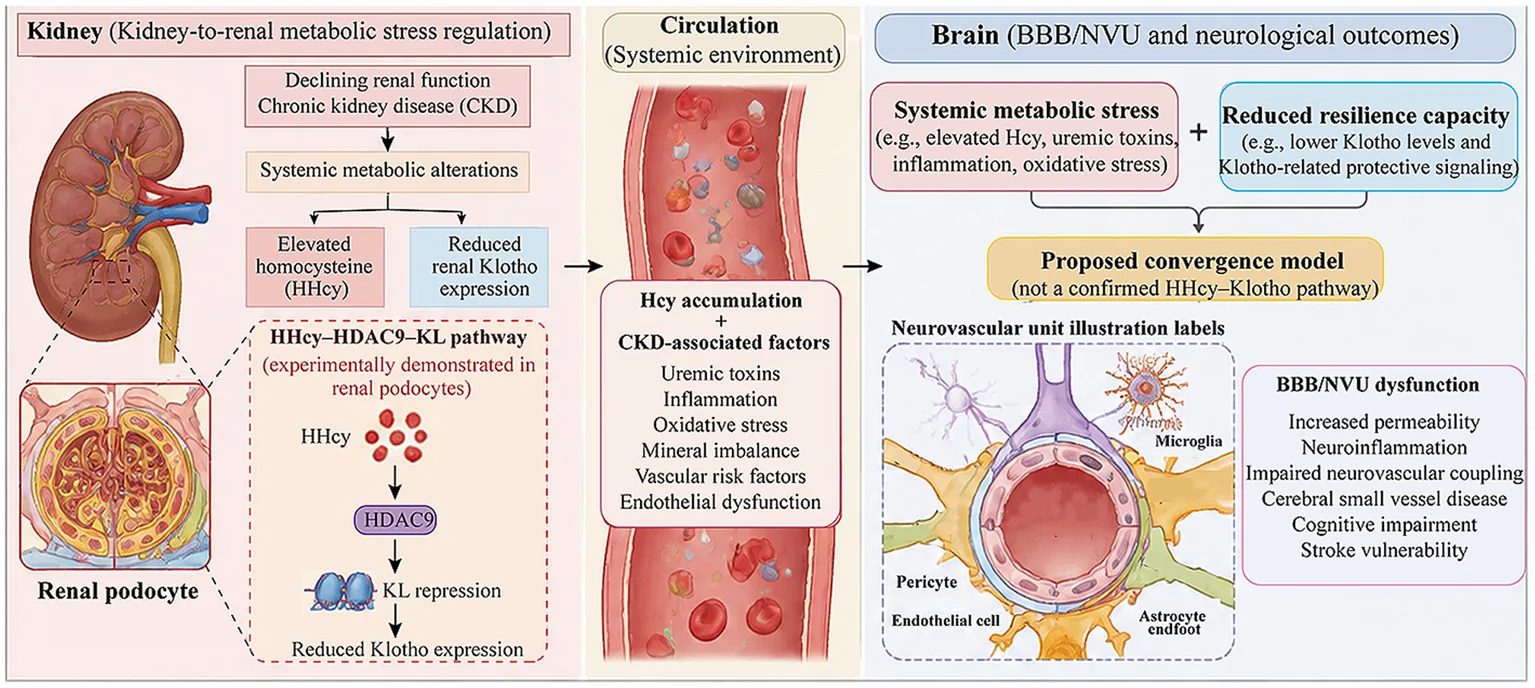
Kidney-to-brain metabolic stress–resilience framework: Established mechanisms and proposed pathways linking HHcy, Klotho, and neurovascular dysfunction. This conceptual framework illustrates how kidney dysfunction, systemic metabolic stress, and reduced resilience capacity may interact to influence neurovascular vulnerability. Chronic kidney disease (CKD) promotes systemic metabolic alterations, including elevated homocysteine (HHcy) and reduced renal Klotho expression, which contribute to an increased systemic stress burden. The HHcy–HDAC9–KL repression pathway represents experimentally demonstrated renal-specific evidence, in which HHcy induces HDAC9 activation and suppresses KL expression in renal podocytes, leading to reduced Klotho expression. However, this mechanism should not be interpreted as an established HHcy–Klotho pathway in cerebral endothelial cells or other neurovascular unit (NVU) components. Following circulation, elevated Hcy and CKD-associated factors, including uremic toxins, inflammation, oxidative stress, mineral imbalance, vascular risk factors, and endothelial dysfunction, may contribute to cerebral endothelial stress and BBB/NVU impairment. In this framework, systemic metabolic stress and reduced Klotho-related resilience are proposed to converge at the cerebral endothelium–BBB–NVU interface, potentially influencing neurovascular dysfunction, cerebral small vessel disease, cognitive impairment, and stroke vulnerability. Importantly, the BBB/NVU-related interactions shown in this model represent a proposed mechanistic framework rather than a confirmed HHcy–Klotho molecular pathway in brain tissues.

## 2 Literature search and evidence evaluation

This review was conducted as a narrative synthesis to develop a conceptual framework linking hyperhomocysteinemia, Klotho biology, kidney dysfunction, and neurovascular vulnerability. Relevant literature was identified through searches of PubMed, Web of Science, and Scopus. Publications available from database inception to December 2025 were considered. The search strategy included combinations of terms related to homocysteine or hyperhomocysteinemia (“homocysteine,” “hyperhomocysteinemia,” “one-carbon metabolism”), Klotho (“Klotho,” “α-Klotho,” “KL”), kidney dysfunction (“chronic kidney disease,” “renal dysfunction”), and neurological or vascular outcomes (“blood–brain barrier,” “neurovascular unit,” “cerebral small vessel disease,” “cognitive impairment,” and “stroke”). Studies were included if they provided experimental, clinical, or translational evidence relevant to the proposed kidney–circulation–BBB/NVU–brain axis. We mainly considered human studies, experimental studies exploring underlying mechanisms, and recent reviews that provided important background information. Studies without direct relevance to Hcy metabolism, Klotho biology, renal dysfunction, or neurological outcomes were not considered. Given the diversity of available evidence, including differences in study design, experimental models, and Klotho measurement compartments, a formal quantitative grading of evidence was not performed. Instead, the evidence was evaluated based on study design, consistency among findings, biological plausibility, and the extent of available mechanistic support. Throughout the review, established findings were distinguished from preliminary or hypothesis-generating observations, particularly when discussing the potential relationship between HHcy and Klotho in brain-related tissues. Because the proposed framework integrates evidence from multiple experimental and clinical settings, the strength and interpretation of evidence vary according to study type. Table 1 summarizes the major evidence categories supporting different components of the framework and highlights their respective contributions and limitations.

**Table 1:** Summary of evidence supporting the HHcy–Klotho stress–resilience framework.

Evidence category	Main evidence source	Key findings relevant to the framework	Contribution to the model	Current limitations	References
Human interventional studies	Hcy-lowering trials in cardiovascular disease or CKD populations	Lowering plasma Hcy can modify one-carbon metabolism and Hcy burden, but neurological and cardiovascular benefits remain inconsistent across populations	Provides information on whether modifying Hcy exposure can alter clinical outcomes	Limited studies directly evaluate BBB/NVU outcomes; no studies have determined whether Klotho status modifies the response to Hcy-lowering therapy	Lee et al., 2024; Yang et al., 2024; Berg et al., 2025; Lin et al., 2026
Human observational studies	CKD cohorts, stroke cohorts, aging and cognitive studies	Elevated Hcy is associated with cognitive impairment, cerebrovascular disease, and adverse neurological outcomes; Klotho-related measures are associated with selected vascular and cognitive phenotypes	Supports the clinical relevance of Hcy-associated stress and Klotho-related resilience as potential determinants of brain vulnerability	Associations do not establish causality; findings may be influenced by renal function, aging, inflammation, and vascular risk factors	Zuliani et al., 2024; Czaplicki et al., 2026; Zhang et al., 2025; Shi et al., 2024; Zhou et al., 2025
Human neuroimaging studies	MRI studies assessing BBB integrity, cerebral perfusion, neurovascular coupling, and CSVD markers	CKD and systemic metabolic abnormalities are associated with BBB disruption, altered cerebral perfusion, and CSVD-related changes	Provides intermediate phenotypes linking peripheral dysfunction with cerebral microvascular injury	Few longitudinal studies simultaneously measure Hcy, Klotho, renal function, neurovascular imaging, and cognition	Bobot et al., 2024; Hu et al., 2024; Song et al., 2024b; Bai et al., 2025; Jiang et al., 2025
Genetic evidence	KL variants and genetic determinants of Hcy metabolism	Genetic variation affecting Klotho biology or Hcy metabolism may influence aging-related and vascular phenotypes	Provides additional biological plausibility for the involvement of Hcy and Klotho pathways	Genetic associations do not demonstrate direct HHcy–Klotho interaction or establish tissue-specific mechanisms	Driscoll et al., 2024; Holmes et al., 2024
Animal studies	CKD models, HHcy models, Klotho deficiency or supplementation models	HHcy promotes oxidative stress, mitochondrial dysfunction, inflammation, endothelial dysfunction, BBB-related injury; Klotho regulates antioxidant, mitochondrial, inflammatory pathways	Provides mechanistic evidence supporting interactions between metabolic stress and resilience pathways	Species differences and incomplete representation of human CKD complexity limit direct translation	Zimmermann et al., 2024; Liu et al., 2024b; Zhu et al., 2024; Xu et al., 2025
*In vitro* studies	Endothelial cells, podocytes, neural and glial cell models	HHcy affects endothelial function, mitochondrial homeostasis, inflammatory signaling, and barrier integrity; HHcy suppresses KL expression through HDAC9-dependent regulation in renal podocytes	Defines potential cellular mechanisms underlying HHcy-associated injury and Klotho-related protection	Direct evidence in cerebral endothelial cells or multicellular NVU models remains limited	Liu et al., 2023; Waithe et al., 2025; Zhou et al., 2026; Chatterjee et al., 2024
Previous reviews and conceptual studies	Reviews of CKD–brain axis, HHcy biology, Klotho signaling, and CSVD mechanisms	Summarize independent evidence domains related to renal dysfunction, vascular injury, metabolism, and brain aging	Provides background evidence supporting integration of previously separate biological pathways	Existing reviews generally focus on individual pathways and do not directly test the proposed HHcy–Klotho stress–resilience framework	Edmonston et al., 2024a; Kanbay et al., 2024a; Kanbay et al., 2024b; Capasso et al., 2025

## 3 HHcy and Klotho in kidney-to-brain communication: metabolic stress and resilience

### 3.1 HHcy as a renal dysfunction-associated systemic metabolic stressor

Hcy is involved in both methionine recycling and the transsulfuration pathway. Its metabolism depends on folate and vitamin B12 for remethylation and on vitamin B6 for transsulfuration. The balance between S-adenosylmethionine and S-adenosylhomocysteine also affects methylation, redox balance, and sulfur-amino-acid metabolism. Therefore, a high plasma Hcy level may reflect broader changes in methyl-group metabolism and antioxidant capacity, rather than a single abnormality in amino-acid metabolism (Joshi and Jadavji, 2024). Plasma Hcy alone does not show all of these biological changes. The transsulfuration pathway connects Hcy metabolism with the production of cysteine, glutathione, and hydrogen sulfide. If this pathway is disturbed, antioxidant defense and redox balance may also change, even when the effect on Hcy concentration is limited (Flori et al., 2025). Abnormal Hcy metabolism may also lead to the formation of Hcy-thiolactone and N-homocysteinylated proteins. These metabolites have been associated with endothelial damage, protein modification, inflammation, and thrombosis (Jakubowski and Witucki, 2025).

This metabolic context is also relevant to the effects of HHcy on the brain. Cerebral blood vessels are exposed to higher levels of Hcy, but the disturbance may extend beyond Hcy itself. Changes in one-carbon and sulfur-amino-acid metabolism may also affect the cerebral vasculature. Renal dysfunction can contribute to these changes, although the kidney is not the source of Hcy. Hcy is produced through methionine metabolism throughout the body, while kidney function strongly affects its level in the blood. As kidney function declines, plasma Hcy levels increase. Therefore, HHcy is particularly common in patients with advanced CKD (Chen et al., 2025). Reduced filtration is unlikely to explain the entire association because CKD also alters vitamin handling, inflammatory status, oxidative balance, protein metabolism, and metabolic clearance. For this reason, HHcy in CKD is best interpreted as a systemic metabolic disturbance associated with renal dysfunction.

Once elevated in the circulation, however, Hcy is directly presented to the luminal surface of cerebral endothelial cells, providing a biologically plausible route through which renal and systemic metabolic dysfunction may be translated into cerebral microvascular stress. Moreover, the metabolic heterogeneity underlying HHcy may partly explain why similar total Hcy concentrations can be associated with different vascular phenotypes and why biochemical lowering of plasma Hcy does not necessarily reverse established neurovascular injury. In this sense, HHcy is useful within the kidney-to-brain continuum not only as a vascular risk marker, but also as a representative component of the systemic metabolic and vascular stress burden to which the cerebral microvasculature is exposed. The relationship between renal dysfunction and HHcy may also operate in both directions. Impaired renal function promotes Hcy accumulation, whereas experimental HHcy can itself injure renal vascular and cellular compartments. The clearest molecular link currently reported between HHcy and Klotho comes from renal podocytes. In renal podocytes, HHcy increased HDAC9 expression, reduced H3K9 acetylation at the KL promoter, lowered Klotho transcription, and worsened podocyte injury (Liu et al., 2023). This finding shows that Hcy can regulate Klotho in a specific renal cell type. It is still unclear whether the same mechanism occurs in cerebral endothelial cells, other NVU cells, or neural cells. Current evidence also does not show that reduced Klotho is responsible for the effects of HHcy on the brain. These changes should be considered in the wider context of CKD. Impaired kidney function is commonly associated with uremic toxin accumulation, inflammation, mineral imbalance, oxidative stress, and altered blood flow, all of which may affect the cerebral circulation. Experimental CKD, for example, has been reported to increase BBB permeability and activate inflammatory signaling in microglia (Zimmermann et al., 2024). HHcy is therefore more appropriately considered one part of the systemic changes associated with CKD, rather than an independent renal neurotoxin.

### 3.2 α-Klotho as a kidney-linked candidate resilience factor

In the proposed kidney-to-brain framework, HHcy mainly represents a component of systemic metabolic and vascular stress. It is associated with oxidative, endothelial, and inflammatory injury, but the consequences of HHcy may also depend on the capacity of tissues to maintain homeostasis and respond to stress. In this context, Klotho is considered a candidate factor contributing to biological resilience. Klotho-related resilience is used here as a conceptual biological construct reflecting the ability of renal, vascular, and neural systems to preserve function under metabolic stress. It does not represent a single measurable parameter and should not be directly inferred from circulating soluble Klotho concentration alone.

α-Klotho is a transmembrane protein with an established role in fibroblast growth factor 23 (FGF23) signaling. Proteolytic cleavage of membrane-bound Klotho generates soluble forms detectable in blood and urine. The kidney is a major site of Klotho expression, and CKD is commonly associated with reduced renal Klotho expression and disruption of the FGF23–Klotho system (Edmonston et al., 2024a; Kanbay et al., 2024a; Kanbay et al., 2024b). This relationship with renal physiology supports the inclusion of Klotho in a kidney-to-brain model. However, Klotho measurements from different compartments, including renal tissue, blood, urine, and CNS, represent distinct biological information. A reduction in circulating Klotho should therefore not be interpreted as direct evidence of reduced brain Klotho activity.

Within this framework, peripheral Klotho may influence brain vulnerability without requiring direct transport of kidney-derived Klotho into the brain. Reduced renal Klotho expression may coexist with oxidative stress, inflammation, endothelial dysfunction, mineral dysregulation, and vascular abnormalities, creating a systemic environment that may affect cerebral perfusion and neurovascular health. Experimental and translational studies support Klotho-related antioxidant, mitochondrial, endothelial, and anti-inflammatory effects, whereas some human studies have associated higher Klotho-related measures with more favorable cognitive or brain-aging phenotypes (Liang et al., 2024; Villacampa et al., 2024; Xu et al., 2025; Czaplicki et al., 2026). These findings support Klotho as a candidate resilience factor, but they do not establish circulating Klotho as a validated causal biomarker or demonstrate that neurological effects require substantial passage of soluble Klotho across an intact BBB.

The interpretation of Klotho biology depends strongly on the compartment being measured. Renal Klotho expression primarily reflects local kidney regulation and may influence systemic metabolic stress through changes in renal function, mineral metabolism, and vascular homeostasis. Circulating soluble Klotho is currently the most accessible measurement in human studies and may provide a partial indication of systemic Klotho availability, however, it cannot directly represent tissue-specific Klotho activity because circulating levels do not necessarily correspond to renal tissue Klotho expression, urinary Klotho, CSF Klotho, or neural Klotho activity (Edmonston et al., 2024b; Martín-Vírgala et al., 2024). Urinary Klotho may reflect renal tubular alterations, whereas CSF and locally produced CNS Klotho may provide more relevant information regarding brain-related Klotho processes. Klotho is also expressed within the CNS, particularly in the choroid plexus and certain neural cell types, where it may contribute to CSF regulation, inflammatory control, and tissue maintenance during aging (Do et al., 2026). The association between lower CSF Klotho and choroid plexus enlargement, together with experimental evidence from local Klotho manipulation studies, further supports the distinction between peripheral and CNS Klotho biology (Sacchi et al., 2025; Fanaei-Kahrani et al., 2026).

Although the present framework primarily focuses on the cerebral endothelium–BBB–NVU interface, the choroid plexus and blood–CSF barrier may represent an additional brain-facing pathway in kidney-to-brain communication. This pathway may be particularly relevant to CNS Klotho biology because CSF Klotho and choroid plexus-derived Klotho may reflect local regulatory processes that are not captured by circulating or renal Klotho measurements. Future studies are needed to clarify how peripheral metabolic stress interacts with local CNS Klotho regulation at this interface.

Therefore, within the stress–resilience framework, different Klotho compartments should be interpreted as reflecting different aspects of Klotho biology rather than interchangeable measures. No currently available Klotho measurement fully captures this multidimensional concept in humans. As illustrated in Figure 2, CKD-associated HHcy and reduced Klotho-related activity represent interacting dimensions of increased systemic stress and reduced protective capacity rather than a simple linear pathway. Their potential convergence is proposed to occur mainly through changes in the systemic vascular environment and the cerebral endothelium–BBB–NVU interface.

**Figure 2:**
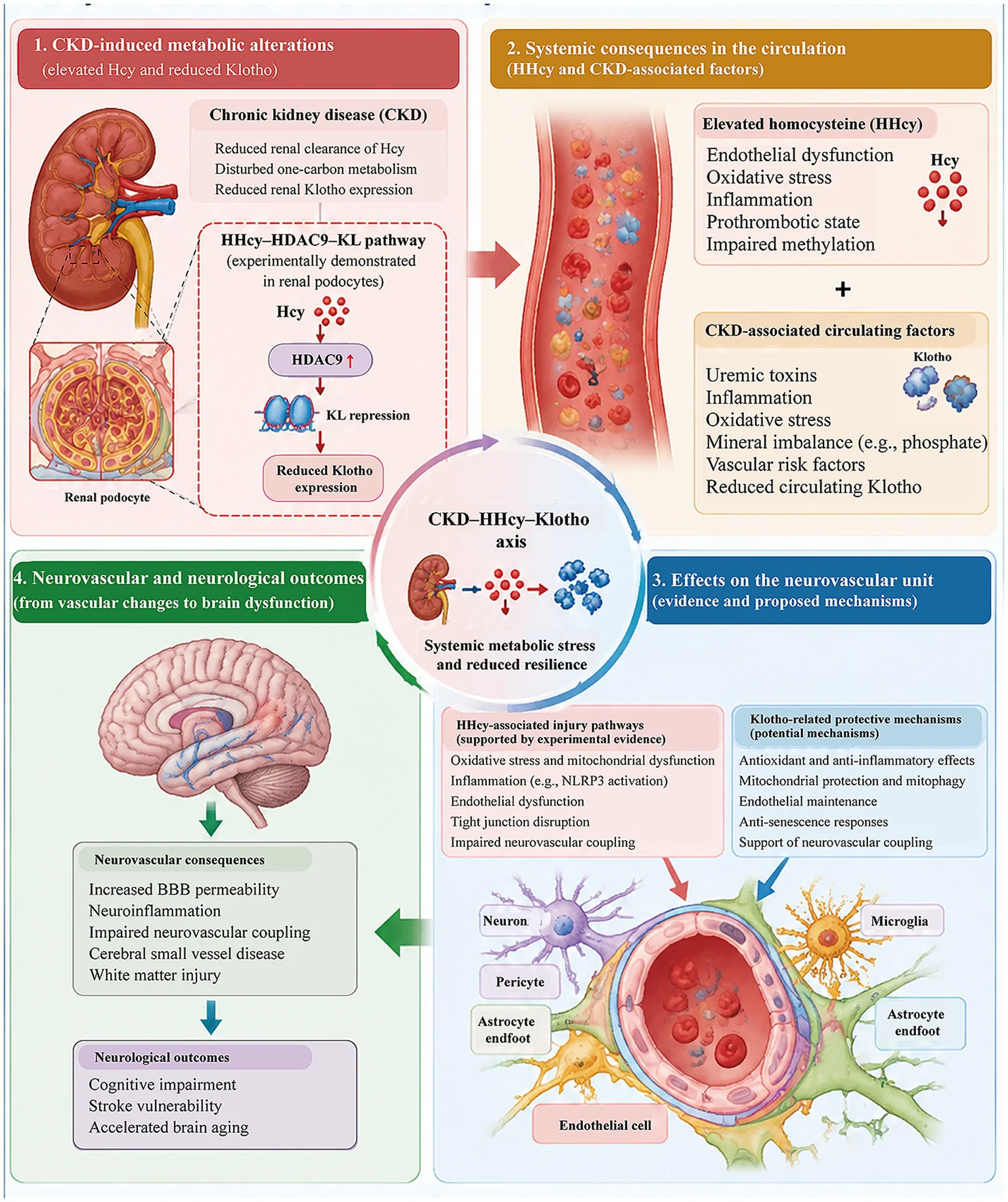
The CKD–HHcy–Klotho stress–resilience framework: From systemic metabolic alterations to neurovascular vulnerability. This schematic illustrates the proposed relationship between chronic kidney disease (CKD)-associated metabolic disturbances, hyperhomocysteinemia (HHcy), Klotho-related resilience, and neurovascular vulnerability. CKD contributes to systemic metabolic alterations through impaired renal clearance of homocysteine (Hcy), disruption of one-carbon metabolism, and reduced renal Klotho expression. The HHcy–HDAC9–KL repression pathway represents an experimentally demonstrated renal-specific mechanism in podocytes, in which HHcy induces HDAC9 activation and suppresses KL expression, leading to reduced Klotho expression. This renal mechanism should not be interpreted as evidence of an identical HHcy-mediated Klotho repression pathway in cerebral endothelial or neurovascular unit (NVU) cells. Circulating Hcy elevation and CKD-associated systemic factors, including uremic toxins, inflammation, oxidative stress, mineral imbalance, and vascular risk factors, may contribute to endothelial dysfunction and increased neurovascular stress. In parallel, reduced Klotho availability or impaired Klotho-related protective signaling may represent a reduced resilience state that could influence tissue responses to metabolic stress. At the neurovascular unit (NVU), HHcy-associated injury pathways, including oxidative stress, mitochondrial dysfunction, inflammation, endothelial dysfunction, tight junction disruption, and impaired neurovascular coupling, may promote blood–brain barrier (BBB) dysfunction and contribute to cerebral small vessel disease, cognitive impairment, and stroke vulnerability. The Klotho-related mechanisms shown in this model represent potential protective pathways, including antioxidant responses, mitochondrial maintenance, anti-inflammatory effects, endothelial preservation, and anti-senescence responses. These mechanisms are supported mainly by experimental studies in renal, vascular, and neural systems, whereas direct evidence demonstrating that Klotho modifies HHcy-induced BBB/NVU injury remains limited. Therefore, this figure represents a conceptual stress–resilience framework integrating established mechanisms and proposed biological convergence rather than a confirmed linear CKD–HHcy–Klotho pathway.

## 4 Neurovascular mechanisms translating HHcy-associated stress and Klotho-related resilience into brain vulnerability

### 4.1 Cerebral endothelium and BBB as primary neurovascular interfaces

The possible link between systemic HHcy and brain injury can first be considered at the cerebral blood vessels. Brain endothelial cells are constantly exposed to metabolites and inflammatory signals in the blood. Because brain endothelial cells are directly exposed to circulating Hcy, Hcy can influence the cerebral environment without first accumulating in neurons. Experimental findings support this vascular route. At moderate levels, Hcy has been reported to impair endothelial cell migration and proliferation, alter mitochondrial respiration, and induce adaptive stress responses rather than causing only acute cytotoxicity (Chatterjee et al., 2024). In human iPSC-derived endothelial models, MTHFR-associated disturbances in Hcy metabolism have also been linked to abnormal mitochondrial morphology, defective mitophagy, and endothelial dysfunction (Yu et al., 2025). These findings place cerebral endothelial metabolism and repair upstream of at least some of the neurological consequences associated with HHcy.

Experimental BBB studies further support an endothelial route. Hcy increases permeability in human brain microvascular endothelial monolayers and reduces ZO-1, while inhibition of the NLRP3 inflammasome partly attenuates this response (Waithe et al., 2025). Other models have linked HHcy with cerebral microcirculatory abnormalities, adhesion-molecule expression, loss of tight-junction proteins, and endoplasmic-reticulum stress (Zhang et al., 2026). Hcy can also enhance amyloid-β-associated cerebral endothelial apoptosis and barrier dysfunction (Carey et al., 2024). Taken together, these observations favor a direct effect of circulating Hcy on the cerebral endothelial barrier, although they do not exclude additional actions in neural or glial cells.

Endothelial dysfunction can then influence the wider NVU. Reduced nitric-oxide availability, impaired vasoreactivity, leukocyte adhesion, prothrombotic signaling, defective vascular repair, and increased barrier permeability alter the environment encountered by pericytes, astrocytic endfeet, microglia, and neurons. CKD provides a relevant clinical setting for this process because severe renal dysfunction may be accompanied by BBB damage and cognitive impairment (Bobot et al., 2024). HHcy, however, is unlikely to explain this relationship on its own. In CKD, elevated Hcy occurs alongside uremic, inflammatory, mineral, metabolic, and hemodynamic disturbances. Together, these abnormalities may increase the vulnerability of the cerebral microvasculature to injury.

### 4.2 Oxidative, mitochondrial, and epigenetic mechanisms of stress-resilience convergence

Redox balance and mitochondrial function may be important points of overlap between HHcy-related injury and Klotho-related protection. HHcy can disturb sulfur-amino-acid metabolism, mitochondrial respiration, endoplasmic-reticulum function, and nitric-oxide signaling. In experimental brain models, HHcy has also been associated with impaired complex-I activity, changes in NAD+/Sirt1 signaling, increased mitochondrial ROS production, abnormal mitophagy, synaptic dysfunction, and cognitive impairment (Chai et al., 2025; Ramires Júnior et al., 2025). These findings suggest that the biological effect of sustained HHcy is broader than direct ROS generation alone; mitochondrial dysfunction, redox imbalance, organellar stress, and defective repair can become mutually reinforcing.

In contrast to the stress burden associated with HHcy, Klotho-related mechanisms may contribute to the resilience side of this balance. Exogenous Klotho can enhance FOXO3a/catalase-related antioxidant responses in astrocytes and proteostatic capacity in neurons, while renal and peripheral experimental models implicate Klotho in mitophagy and Nrf2/HO-1-associated protection against oxidative stress, mitochondrial dysfunction, and cellular senescence (Orellana et al., 2023; Zhu et al., 2024; Xu et al., 2025). Across experimental vascular and peripheral-cell models, Klotho has also been shown to modulate endothelial stress responses, oxidative and inflammatory signaling, mitochondrial homeostasis, and cellular senescence. These effects have not been demonstrated uniformly in cerebral endothelial cells and should therefore be considered candidate mechanisms through which Klotho status might modify neurovascular vulnerability rather than confirmed components of a unified HHcy-Klotho pathway. Importantly, the protective effects attributed to Klotho have been reported in different experimental models and should not be interpreted as direct evidence that Klotho protects cerebral endothelial cells from HHcy-induced injury. Klotho-related effects on oxidative stress, mitochondrial function, inflammation, endothelial responses, and cellular senescence have been observed in renal cells, peripheral vascular models, astrocytes, neurons, ischemic models, and inflammatory disease models. These studies support the possibility that Klotho contributes to stress resistance, but whether these mechanisms occur in HHcy-exposed cerebral endothelial cells or other BBB/NVU components remains unclear. To better define the available evidence, Table 2 summarizes the major experimental models, cell types, Klotho interventions, and reported outcomes relevant to Klotho-related protective mechanisms. In particular, direct evidence that Klotho specifically rescues Hcy-induced injury in cerebral endothelial cells remains limited.

**Table 2:** Experimental evidence supporting Klotho-related protective mechanisms and relevance to HHcy-associated neurovascular injury.

Tissue/System	Cell type or model	Klotho intervention	Stress condition	Main endpoint	Relevance to HHcy–BBB/NVU framework	Evidence limitation
Kidney	Podocytes	Klotho restoration/overexpression	HHcy model	Reduced oxidative stress, improved podocyte injury	Supports HHcy–Klotho interaction in renal tissue	Not a brain or BBB model
Kidney	Renal tubular cells	Klotho supplementation	CKD-related stress	Reduced inflammation and oxidative injury	Supports renal resilience role of Klotho	Does not establish brain effects
Peripheral vascular system	Endothelial cells	Exogenous Klotho	Oxidative or vascular stress	Improved endothelial function and redox balance	Supports endothelial protective capacity	Not cerebral endothelial cells exposed to Hcy
Astrocytes	Astrocyte models	Klotho treatment	Oxidative stress	Increased antioxidant response (FOXO3a/catalase pathway)	Supports neural stress resistance	Does not represent BBB interface
Neurons	Neuronal models	Klotho administration	Aging/metabolic stress	Improved mitochondrial and proteostatic function	Supports neuronal resilience	Limited relevance to vascular entry point
Ischemic models	Animal ischemia models	Klotho overexpression or administration	Cerebral ischemic injury	Reduced inflammation, improved tissue protection	Supports protective role under brain injury conditions	Not specific to HHcy
Inflammatory CNS models	EAE/inflammatory models	Klotho-secreting cells	Neuroinflammation	Reduced BBB permeability and inflammatory markers	Provides indirect BBB-related evidence	Disease model differs from HHcy

Epigenetic regulation offers a useful mechanistic clue, although the evidence is currently tissue specific. Because Hcy metabolism is linked directly to S-adenosylmethionine and S-adenosylhomocysteine, persistent disturbance of this pathway can alter the biochemical environment required for methylation-dependent regulation. In renal podocytes, HHcy represses KL through HDAC9-dependent chromatin changes (Liu et al., 2023). Brain microvascular endothelial cells show a different Hcy-sensitive epigenetic response: Cacna1c/Cav1.2-related signaling has been linked to altered calcium handling, ROS production, degradation of tight-junction components, BBB disruption, and cognitive impairment (Zhou et al., 2026). The two observations establish epigenetic effects of HHcy in both renal and cerebral vascular settings, but they do not yet demonstrate a common HHcy-Klotho epigenetic pathway. Direct assessment of KL promoter methylation, histone acetylation, chromatin accessibility, and Klotho expression in cerebral endothelial and other NVU cell types is therefore required.

Epigenetic change may also help explain how an intermittent metabolic disturbance becomes associated with more persistent vascular vulnerability. Human observations are consistent with this possibility but remain associative. Among individuals with mild cognitive impairment, higher Hcy has been associated with faster epigenetic aging (Holmes et al., 2024). This does not establish a direct causal effect of Hcy on a genome-wide aging program, but it argues for considering epigenetic state alongside conventional plasma measurements. On the resilience side, KL-VS heterozygosity in cognitively unimpaired older adults has been associated with smaller age-related changes in CSF markers of inflammation, neurodegeneration, and synaptic dysfunction (Driscoll et al., 2024). These findings also reinforce the need to distinguish Klotho genotype from circulating Klotho, renal expression, and locally produced CNS Klotho.

### 4.3 Neuroinflammatory amplification within the NVU

The NLRP3-dependent barrier response described above provides one mechanistic bridge from endothelial injury to neuroinflammatory amplification. HHcy activates redox-sensitive inflammatory pathways, and at the brain endothelial barrier, NLRP3/caspase-1 signaling contributes to Hcy-induced hyperpermeability (Ramires Júnior et al., 2025; Waithe et al., 2025). CKD creates an additional inflammatory background in which this response may occur: experimental renal dysfunction can increase BBB permeability and activate microglial inflammasome pathways that contribute to neuronal dysfunction (Zimmermann et al., 2024). A high-Hcy state occurring within this CKD-associated milieu may therefore have a greater cerebral effect than the same Hcy burden in a vascular system with preserved homeostatic and reparative capacity.

Klotho intersects with several inflammatory and cellular stress-response pathways. Experimental evidence implicates Klotho in the regulation of NF-κB, Toll-like receptor, MAPK, renin angiotensin, and TGF-βassociated signaling, and Klotho contributes to ischemic tolerance in models involving NLRP3-associated pyroptosis (Liu et al., 2024b; Zimmermann et al., 2024). Klotho-secreting mesenchymal stromal cells have also reduced BBB permeability and inflammatory adhesion molecules in experimental autoimmune encephalomyelitis (Maleki et al., 2025). These findings support the possibility that Klotho-related mechanisms can modify inflammatory and barrier responses. However, they do not establish that circulating renal Klotho directly reaches the cerebral endothelium or specifically neutralizes Hcy-induced BBB injury.

This anti-inflammatory dimension is particularly relevant to organ-to-brain communication because peripheral disturbances do not need to enter the brain parenchyma directly to influence neural homeostasis. Circulating metabolites, inflammatory mediators, and systemic vascular signals can first alter cerebral endothelial activation, leukocyte-endothelial interactions, and systemic or perivascular immune tone. Klotho has been linked to restraint of inflammatory signaling across aging-related contexts, including pathways involving NF-κB, cytokine production, and immune-cell activation (Prud'homme and Wang, 2024). Therefore, the reduced Klotho does not function as a single inflammatory trigger. Instead, diminished Klotho-related resilience may lower the capacity of vascular and neural systems to constrain the inflammatory consequences of HHcy-associated and CKD-associated stress, thereby facilitating leukocyte adhesion, inflammasome activation, barrier leakage, and secondary glial responses. Direct testing in brain endothelial and multicellular NVU models will be required to distinguish a true modifying effect of Klotho from parallel associations driven by renal dysfunction, aging, and systemic inflammation.

The NVU therefore provides the multicellular context in which a blood-facing endothelial disturbance can be translated into broader cerebral dysfunction. Increased BBB permeability and endothelial inflammatory signaling can alter pericyte function, disrupt astrocyte-endothelial communication, promote microglial activation, impair capillary flow and neurovascular coupling, and compromise neuronal substrate delivery. Persistent oxidative and mitochondrial stress may further promote endothelial senescence, reduce reparative capacity and generate a sustained inflammatory environment that can persist beyond the initiating metabolic insult (Jia et al., 2024; Lochhead et al., 2024; Novo et al., 2024; Kremer et al., 2025). In this formulation, HHcy contributes to the initiating and propagating burden of metabolic and neurovascular stress, whereas reduced Klotho-related resilience may diminish the capacity of vascular and neural systems to contain, compensate for, or recover from that stress. The resulting pathology is therefore better conceptualized as a set of self-reinforcing neurovascular loops in which metabolic stress, impaired resilience, barrier dysfunction, and neuroinflammation amplify one another, rather than as a single unidirectional HHcy-Klotho molecular cascade (Figure 3).

**Figure 3:**
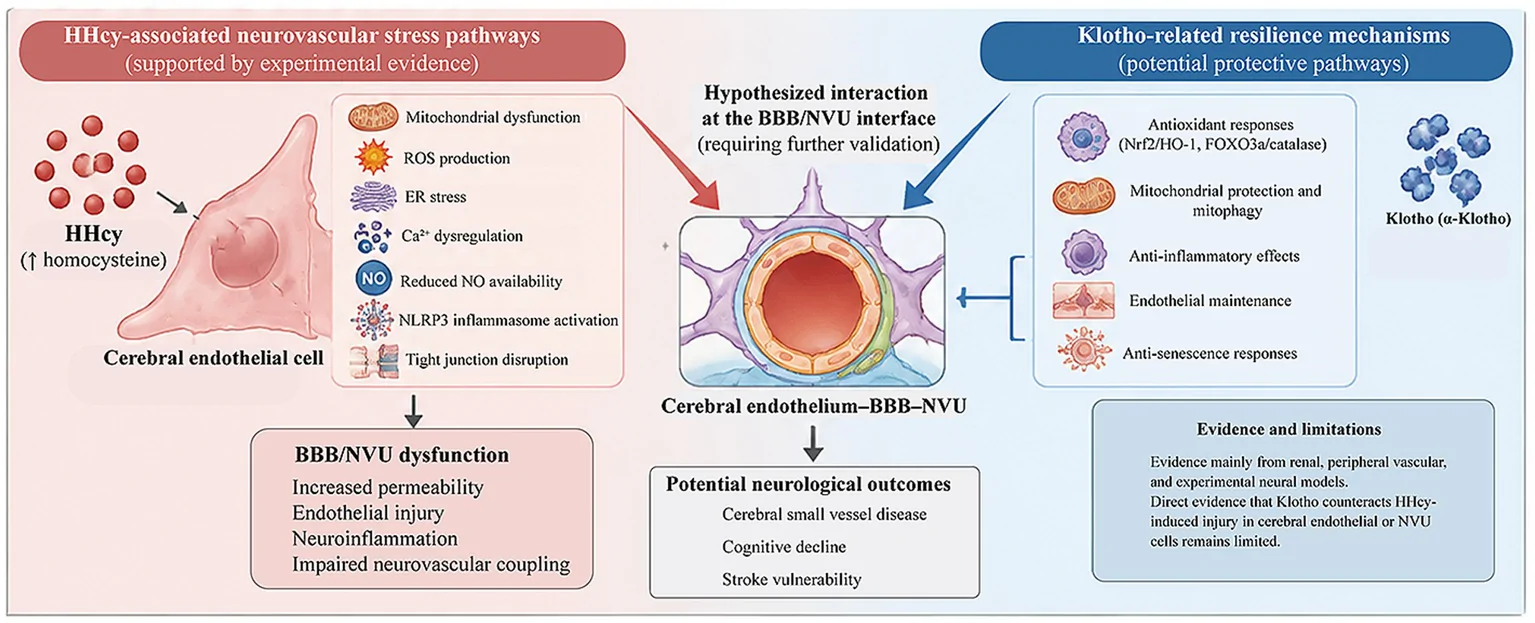
Proposed convergence of HHcy-associated stress and Klotho-related resilience at the cerebral endothelium–BBB–NVU interface: Current evidence and unresolved mechanisms. This schematic summarizes the potential interaction between HHcy-associated neurovascular stress pathways and Klotho-related resilience mechanisms at the cerebral endothelium–blood–brain barrier (BBB)–neurovascular unit (NVU) interface. HHcy has been associated with multiple cellular stress responses relevant to neurovascular injury, including mitochondrial dysfunction, reactive oxygen species (ROS) production, endoplasmic reticulum (ER) stress, calcium dysregulation, reduced nitric oxide (NO) availability, NLRP3 inflammasome activation, and tight junction disruption. These processes may contribute to increased BBB permeability, endothelial injury, neuroinflammation, and impaired neurovascular coupling. The Klotho-related resilience arm represents potential protective mechanisms supported mainly by studies in renal, peripheral vascular, and experimental neural systems. These mechanisms include antioxidant responses, mitochondrial protection and mitophagy, anti-inflammatory effects, endothelial maintenance, and anti-senescence responses. However, direct evidence demonstrating that Klotho counteracts HHcy-induced injury in cerebral endothelial cells or NVU components remains limited. Therefore, this figure should be interpreted as a proposed convergence model in which HHcy-associated stress and Klotho-related resilience may influence neurovascular vulnerability, rather than as evidence for an established HHcy–Klotho pathway within the brain. Further mechanistic studies using cerebral endothelial and multicellular NVU models are required to determine whether Klotho directly modifies HHcy-induced neurovascular injury.

## 5 Clinical contexts for testing the HHcy-Klotho stress-resilience framework

### 5.1 CKD as a clinical setting linking BBB dysfunction, neurovascular coupling, and cognition

CKD is well suited to testing the proposed stress-resilience model. As renal function worsens, Hcy levels usually rise and renal Klotho expression falls, while uremic, inflammatory, mineral, vascular, and hemodynamic abnormalities become more pronounced. Cognitive impairment is also frequent in CKD, particularly in attention and executive function (Zhang et al., 2024). In patients with end-stage kidney disease, BBB imaging has shown greater barrier permeability, and this change is associated with poorer cognitive performance (Bobot et al., 2024). These findings show that severe renal dysfunction can coexist with measurable BBB damage, but it does not establish HHcy or Klotho as the main cause. BBB permeability may therefore serve as a useful intermediate measure when studying kidney-to-brain mechanisms.

Any interpretation of Hcy and Klotho in CKD must take the surrounding disease milieu into account. Retained solutes, anemia, mineral abnormalities, arterial stiffness, oxidative stress, chronic inflammation, and altered hemodynamics can all affect cerebral endothelial function and cognition. HHcy and reduced Klotho are therefore mechanistically relevant components of CKD rather than complete explanations for CKD-associated neurological dysfunction. Recent reviews similarly describe cognitive impairment in CKD as the product of interacting vascular, metabolic, inflammatory, and toxin-mediated processes (Capasso et al., 2025). Uremic toxins are of particular interest because their accumulation during renal failure can promote endothelial and inflammatory stress with potential effects on the cerebral circulation (Bossola and Picconi, 2024; Andrews et al., 2025). The clinically important question is therefore not whether HHcy or Klotho alone explains kidney-associated neurological disease, but whether their measurement adds mechanistic, predictive, or stratifying information beyond established indicators of renal and vascular dysfunction. Importantly, high Hcy and low Klotho should not be considered completely independent of CKD severity. Declining renal function can increase plasma Hcy levels, reduce renal Klotho expression, and promote other systemic abnormalities, including uremic toxin accumulation, inflammation, mineral metabolism disturbance, vascular stiffness, and hemodynamic changes. Therefore, these factors may partially reflect the overall burden of kidney disease. However, Hcy and Klotho may also provide different biological information within this shared disease context. Hcy is considered here as a marker of metabolic and vascular stress, whereas Klotho-related measures may reflect a component of renal-linked protective capacity or resilience. The key question is whether combined assessment of Hcy and Klotho provides additional information beyond conventional measures of kidney function, such as eGFR and albuminuria. This hypothesis requires prospective studies using appropriate adjustment models and longitudinal evaluation of BBB/NVU injury, cerebral small-vessel disease, and cognitive outcomes.

Multimodal neuroimaging provides an opportunity to test this question at intermediate stages between systemic dysfunction and overt cognitive impairment. Non-dialysis CKD and end-stage kidney disease have been associated with abnormalities in cerebral perfusion, neurovascular coupling, spontaneous brain activity, and network connectivity, several of which correlate with cognitive performance or the severity of renal impairment (Hu et al., 2024; Song et al., 2024b; Bai et al., 2025; Jiang et al., 2025). Together with BBB permeability, these measures provide intermediate phenotypes that map directly onto the cerebral endothelium-BBB-NVU mechanisms described in Section 3. Prospective studies could therefore determine whether increasing Hcy burden and reduced Klotho-related measures precede or accompany deterioration in BBB integrity, cerebral blood flow, neurovascular coupling, and cognition as renal function declines.

Human data further suggest that renal function may modify the neurological context in which HHcy is expressed. In ischemic stroke, elevated Hcy has shown a stronger association with post-stroke cognitive impairment among individuals with lower eGFR (Zhang et al., 2025). Such observations do not establish biological interaction or mechanistic synergy, but they are consistent with the stress-resilience hypothesis that the cerebral consequences of a given Hcy burden depend on the systemic and vascular environment in which that burden occurs. The same circulating Hcy concentration may therefore not carry equivalent neurological significance across individuals with preserved renal and vascular homeostasis and those with CKD-associated inflammation, endothelial dysfunction, uremia, and reduced reparative capacity.

Experimental CKD has been associated with reduced membrane-bound Klotho in the frontal cortex together with cognitive impairment (Rodríguez-Ortiz et al., 2024). These findings suggest that changes in renal and cerebral Klotho may occur at the same time. However, they do not show that Klotho produced by the kidney is transported directly into the brain. Population studies also support a broader vascular link between the kidney and the brain. In the Framingham Heart Study, impaired kidney function and albuminuria were associated with cognitive disorders and markers of cerebral small-vessel disease (Kelly et al., 2024). These findings are consistent with an organ-to-brain model involving metabolic, inflammatory, and vascular changes. However, they do not show that circulating Klotho is the direct cause of this relationship. CKD therefore provides a useful clinical setting for prospective testing of the proposed mechanism. Longitudinal studies could measure Hcy, Klotho in clearly defined biological compartments, eGFR, albuminuria, uremic and inflammatory markers, vascular risk factors, BBB permeability, cerebral perfusion or neurovascular coupling, and cognitive changes. Such studies could test whether patients with higher Hcy and lower Klotho levels have a stronger association between declining kidney function and cerebral vascular injury. They could also determine whether Klotho changes the effect of Hcy, rather than simply reflecting the severity of kidney disease or biological aging.

### 5.2 CSVD and cognitive impairment as priority downstream phenotypes

Cerebral small-vessel disease (CSVD) is a useful downstream phenotype for this model because it mainly involves the cerebral microvasculature. Current models of CSVD focus on chronic endothelial dysfunction, BBB leakage, impaired cerebrovascular reactivity, oxidative stress, low-grade inflammation, and gradual damage to the NVU (Jia et al., 2024; Lochhead et al., 2024; Kremer et al., 2025). These processes are affected by systemic blood pressure, circulating metabolites, kidney dysfunction, and inflammatory factors. Over time, these systemic changes may contribute to white-matter damage and small-vessel injury.

CSVD also develops over a long period before clear clinical symptoms appear. Changes in autoregulation, endothelial function, BBB integrity, inflammation, oxidative stress, perivascular tissue, and white matter may begin years before dementia or stroke becomes clinically apparent (Hainsworth et al., 2024; Hannawi, 2024). Therefore, BBB permeability, cerebrovascular reactivity, cerebral perfusion, white-matter integrity, white-matter-hyperintensity burden, lacunes, microbleeds, and enlarged perivascular spaces can be useful measures in longitudinal studies. The main question is not only whether HHcy and low Klotho are present in patients with CSVD. It is also important to determine whether systemic stress and biological reserve affect how quickly peripheral changes lead to progressive cerebral microvascular injury.

Observational studies of CSVD provide support for the vascular component of this model. Higher circulating Hcy has been associated with white-matter hyperintensities, microbleeds, enlarged perivascular spaces, lacunes, and cognitive impairment (Ji et al., 2020). Klotho-related observations point in the opposite direction: lower plasma Klotho has been reported in association with vascular dementia, lacunar infarction, leukoaraiosis, and greater CSVD burden or progression (Brombo et al., 2018). These two sets of findings are compatible with a high-Hcy/low-Klotho phenotype, but substantial confounding remains possible. Renal function, age, vascular disease, inflammatory state, and differences in Klotho assays may influence both measurements. Joint measurement is therefore more informative at this stage than assuming a direct Hcy-to-Klotho pathway in cerebral vessels.

Associations with Hcy are also apparent when CSVD is evaluated beyond individual lesion types. In cognitively healthy older adults, higher Hcy has been linked with greater white-matter-hyperintensity burden, cortical and subcortical structural abnormalities, and slower processing speed (Song et al., 2024a). A systematic review and meta-analysis of biomarkers associated with CSVD-related cognitive impairment also identified Hcy, although heterogeneity in disease definition, study design, and biomarker measurement was substantial (Liao et al., 2024). Among patients with type 2 diabetes, higher Hcy has additionally been associated with greater total CSVD burden (Huang et al., 2025), suggesting that the vascular consequences of HHcy may depend partly on the metabolic context in which it occurs. These observations support the use of quantitative CSVD progression and longitudinal cognitive change as outcomes rather than restricting the role of Hcy to overt stroke risk.

Cognitive aging provides a broader but complementary clinical context. Higher Hcy has been associated with progression from mild cognitive impairment to dementia, whereas higher serum Klotho has been associated with more favorable relationships between age-related brain atrophy and cognitive performance in cognitively unimpaired middle-aged and older adults (Zuliani et al., 2024; Czaplicki et al., 2026). These findings are consistent with the proposed distinction between metabolic stress burden and resilience, but they do not demonstrate that Hcy and Klotho interact biologically in humans. Both measures are influenced by or associated with renal function, nutritional status, vascular disease, inflammation, and aging, and circulating Klotho should not be assumed to represent local CNS Klotho activity. For this reason, longitudinal changes in BBB permeability, cerebral perfusion, CSVD burden, brain structure, and cognition are likely to provide more mechanistically informative outcomes than dementia diagnosis alone.

HHcy has been associated with cerebrovascular risk and adverse outcomes in selected populations, whereas lower early serum Klotho has been associated with unfavorable functional outcomes after acute ischemic stroke (Shi et al., 2024; Zhou et al., 2025; Bi et al., 2026). Stroke also provides a useful stress challenge through which pre-existing neurovascular vulnerability can become clinically apparent. The relevant hypothesis is that a high systemic stress burden associated with HHcy, occurring in a setting of reduced Klotho-related resilience and renal or vascular dysfunction, may influence both susceptibility to ischemic injury and the capacity for subsequent neurovascular recovery. This possibility remains to be tested using studies that measure Hcy and Klotho jointly and relate them to renal function, BBB/NVU phenotypes, lesion evolution, and neurological recovery.

An important limitation of current clinical studies is that Hcy and Klotho have usually been investigated separately. Most human studies have examined the relationship of Hcy or Klotho with vascular disease, brain imaging markers, cognitive outcomes, or stroke. Therefore, it remains unknown whether Klotho status changes the association between Hcy levels and neurovascular outcomes. To date, there is limited evidence from human cohorts showing that the relationship between Hcy and BBB/NVU injury, cerebral small-vessel disease, or cognitive decline differs according to Klotho levels. In particular, studies directly testing Hcy × Klotho interaction effects are still lacking.

This point is important because the main hypothesis of the present framework is not simply that elevated Hcy and reduced Klotho are each associated with adverse outcomes. Rather, it proposes that reduced Klotho-related resilience may influence how tissues respond to metabolic stress. Therefore, the high-Hcy/low-Klotho pattern should currently be viewed as a hypothesis-generating model rather than an established clinical phenotype. Future studies should measure Hcy and Klotho together, carefully distinguish different Klotho compartments, and determine whether Klotho modifies the relationship between Hcy burden and longitudinal changes in BBB function, cerebral small-vessel disease, and cognition.

Taken together, current clinical evidence supports individual components of the proposed kidney-circulation-BBB/NVU-brain continuum, but not yet the complete causal sequence (Table 3). Renal dysfunction is associated with cognitive and cerebral microvascular abnormalities; HHcy is associated with BBB-related, CSVD, and cognitive phenotypes; and Klotho-related measures are associated with selected vascular and neurological outcomes. What remains largely missing is longitudinal evidence integrating these components within the same individuals. The key clinical test of the stress-resilience framework is therefore whether combined assessment of Hcy burden and Klotho-related resilience improves prediction or mechanistic explanation of BBB/NVU dysfunction, CSVD progression, and cognitive decline beyond renal function and conventional vascular risk factors alone (Table 4).

**Table 3:** Evidence maps for the proposed kidney-to-brain HHcy–Klotho stress–resilience framework.

Evidence element	Evidence summarized in the review	Evidence type	Framework interpretation	Key caveat
Renal dysfunction → Hcy accumulation	Plasma Hcy rises as renal function declines, and HHcy is common in advanced CKD.	Human clinical/physiologic	Supported component of the kidney-to-brain context	HHcy is a renal dysfunction-associated systemic disturbance, not a kidney-derived signal.
HHcy → renal Klotho repression	In renal podocytes, HHcy increased HDAC9, reduced H3K9 acetylation at the KL promoter, suppressed Klotho transcription, and aggravated injury.	Direct experimental; renal cell	Specific mechanistic HHcy-to-Klotho link established in kidney	Not shown to operate in cerebral endothelial cells, other NVU cells, or as a mediator of brain injury.
Hcy → cerebral endothelial/BBB injury	Hcy impairs endothelial migration/proliferation and mitochondrial function; increases BBB permeability; reduces ZO-1; and engages NLRP3, ER-stress and epigenetic mechanisms.	Experimental brain endothelial/animal	Direct support for the blood-facing cerebral interface	The size and mechanism of effects are model- and context-dependent; this does not establish a Klotho-dependent pathway.
CKD → BBB/NVU dysfunction	Experimental CKD increases BBB permeability and microglial inflammatory signaling; severe kidney disease can coexist with measurable BBB disruption and poorer cognition.	Experimental + human imaging	Supports the kidney–circulation–BBB/NVU–brain continuum	Does not identify HHcy or Klotho as the mediator of CKD-associated brain injury.
Klotho-related protective mechanisms	Experimental studies implicate Klotho in antioxidant, mitochondrial, endothelial, anti-inflammatory, proteostatic and anti-senescent responses.	Experimental/translational	Supports a candidate resilience dimension	Not uniformly demonstrated in cerebral endothelium; direct rescue of Hcy-induced cerebral endothelial injury remains limited.
Klotho compartmentalization	Renal membrane Klotho, urinary Klotho, serum soluble Klotho, CSF Klotho and local CNS Klotho represent related but non-equivalent compartments.	Biological/measurement framework	Core interpretive constraint	Serum soluble Klotho cannot be assumed to mirror renal tissue expression, CSF Klotho or local CNS Klotho activity.
HHcy → CSVD/cognitive phenotypes	Higher Hcy has been associated with white-matter hyperintensities, microbleeds, enlarged perivascular spaces, lacunes, cognitive impairment and MCI-to-dementia progression.	Human observational	Clinically consistent downstream stress signal	Associations are vulnerable to confounding by renal function, age, B-vitamin status, vascular disease and inflammation.
Lower Klotho → vascular/neurologic phenotypes	Lower Klotho-related measures have been associated with vascular dementia, lacunar infarction, leukoaraiosis, greater CSVD burden/progression, stroke outcomes and selected brain-aging phenotypes.	Human observational	Clinically consistent candidate resilience signal	Assay heterogeneity and compartment differences limit causal interpretation.
Hcy × Klotho interaction	The central prediction is that Klotho status modifies the neurovascular consequences of a defined Hcy burden.	Hypothesis/key gap	Decisive test of the proposed stress–resilience framework	No validated interaction model, molecular axis, biomarker ratio, diagnostic cut-off or treatment threshold currently exists.

**Table 4:** Priority clinical contexts and measurements for testing the framework.

Clinical context	Why it is informative	Systemic/Renal measurements	Priority neurovascular or imaging phenotypes	Clinical outcomes/Key covariates
Chronic kidney disease (CKD)	Renal function spans a continuum that can simultaneously alter Hcy burden, Klotho biology, uremic/inflammatory stress, mineral metabolism and hemodynamics.	Hcy; clearly defined Klotho compartment; eGFR; albuminuria; uremic markers; inflammatory markers; B-vitamin status; mineral metabolism; blood pressure.	BBB permeability; cerebral perfusion; neurovascular coupling; spontaneous activity/network connectivity; structural CSVD burden.	Attention/executive function and cognitive trajectory. Key covariates: age, anemia, vascular disease, diabetes, nutrition, inflammation.
Cerebral small-vessel disease (CSVD)	Directly interrogates the same cerebral microvascular interface implicated by the mechanistic framework and provides longitudinal intermediate endpoints.	Hcy; Klotho; renal function; vascular risk; inflammatory markers.	White-matter hyperintensities; lacunes; microbleeds; enlarged perivascular spaces; BBB permeability; cerebrovascular reactivity; perfusion; total CSVD burden.	Processing speed, executive function, cognitive decline. Adjust for age, blood pressure, diabetes, kidney function and vascular disease.
Cognitive aging/MCI	Provides a broader clinical setting in which stress burden, resilience and age-related molecular remodeling can be followed before dementia.	Hcy; Klotho measure and/or KL-VS genotype; eGFR; B-vitamin status; inflammatory markers.	Brain atrophy; white-matter integrity; CSVD burden; optional CSF markers and epigenetic-aging measures.	MCI progression, processing speed, executive function and global cognition. Key covariates: age, renal function, nutrition, vascular disease.
Ischemic stroke	Acts as a vascular stress challenge that may expose pre-existing neurovascular vulnerability and recovery capacity.	Early Hcy; early serum Klotho with explicit assay/compartment definition; eGFR; inflammatory and vascular markers.	Lesion evolution; BBB/NVU phenotypes where feasible; perfusion; quantitative small-vessel disease background.	Post-stroke cognition, functional outcome and recovery. Test whether renal function and Klotho modify the Hcy–outcome association.

## 6 Translational implications

### 6.1 From isolated biomarkers to joint mechanistic stratification

The translational value of Hcy and Klotho may lie less in their use as isolated biomarkers than in the distinct biological dimensions they potentially represent. Hcy is analytically accessible and reflects one-carbon metabolism, nutritional status, renal function, and vascular stress. Klotho-related measures may provide complementary information about renal integrity and biological resilience, although the interpretation of soluble Klotho remains limited by methodological heterogeneity and biological compartmentalization (Edmonston et al., 2024a; Kanbay et al., 2024a). A high-Hcy/low-Klotho pattern may represent a hypothesis-generating stress–resilience model characterized by increased metabolic stress together with potentially reduced Klotho-related resilience. Whether this pattern represents a clinically useful phenotype remains uncertain and requires validation in cohorts with simultaneous Hcy and Klotho measurements and direct assessment of interaction effects (Figure 2). This formulation remains hypothesis-generating: no diagnostic or therapeutic HHcy-Klotho ratio, cut-off, or validated risk score is currently supported by the available evidence.

The Klotho component of such a strategy requires particular caution. A recent systematic review and meta-analysis supports an overall relationship between Klotho-related measures and cognitive or dementia outcomes, but between-study heterogeneity is substantial and most available evidence is observational (Liu et al., 2025). Circulating soluble Klotho measurements are also sensitive to assay platform, sample handling, renal function, and biological compartment (Martín-Vírgala et al., 2024). Serum Klotho should therefore not be assumed to provide a direct quantitative measure of systemic resilience or local CNS Klotho activity. A clinically informative HHcy-Klotho phenotype would require standardized Klotho measurement together with explicit consideration of eGFR, albuminuria, age, mineral metabolism, inflammation, nutritional status, and vascular risk rather than reliance on a simple numerical ratio.

The value of measuring Hcy and Klotho together depends on whether they provide different biological information. A causal framework is therefore needed to distinguish shared determinants from potential independent biological contributions. In this model, CKD severity represents an upstream driver that influences Hcy, Klotho, and multiple systemic abnormalities at the same time. These parallel pathways may converge at the cerebral endothelium and BBB/NVU interface, where the balance between metabolic stress and protective capacity may influence neurological vulnerability (Figure 4). Importantly, the proposed relationships should be interpreted as a conceptual model rather than as a confirmed causal pathway. Hcy may reflect metabolic and vascular stress, while Klotho-related mechanisms may help tissues respond to that stress. Therefore, people with similar Hcy levels may not have the same effects on the brain if their Klotho status is different. This idea can be tested by examining whether Klotho changes the relationship between Hcy and BBB permeability, cerebral perfusion, neurovascular coupling, endothelial activation, CSVD progression, or cognitive decline. Evidence of such effect modification would support the proposed framework more strongly than independent associations of the two biomarkers with the same endpoint.

**Figure 4:**
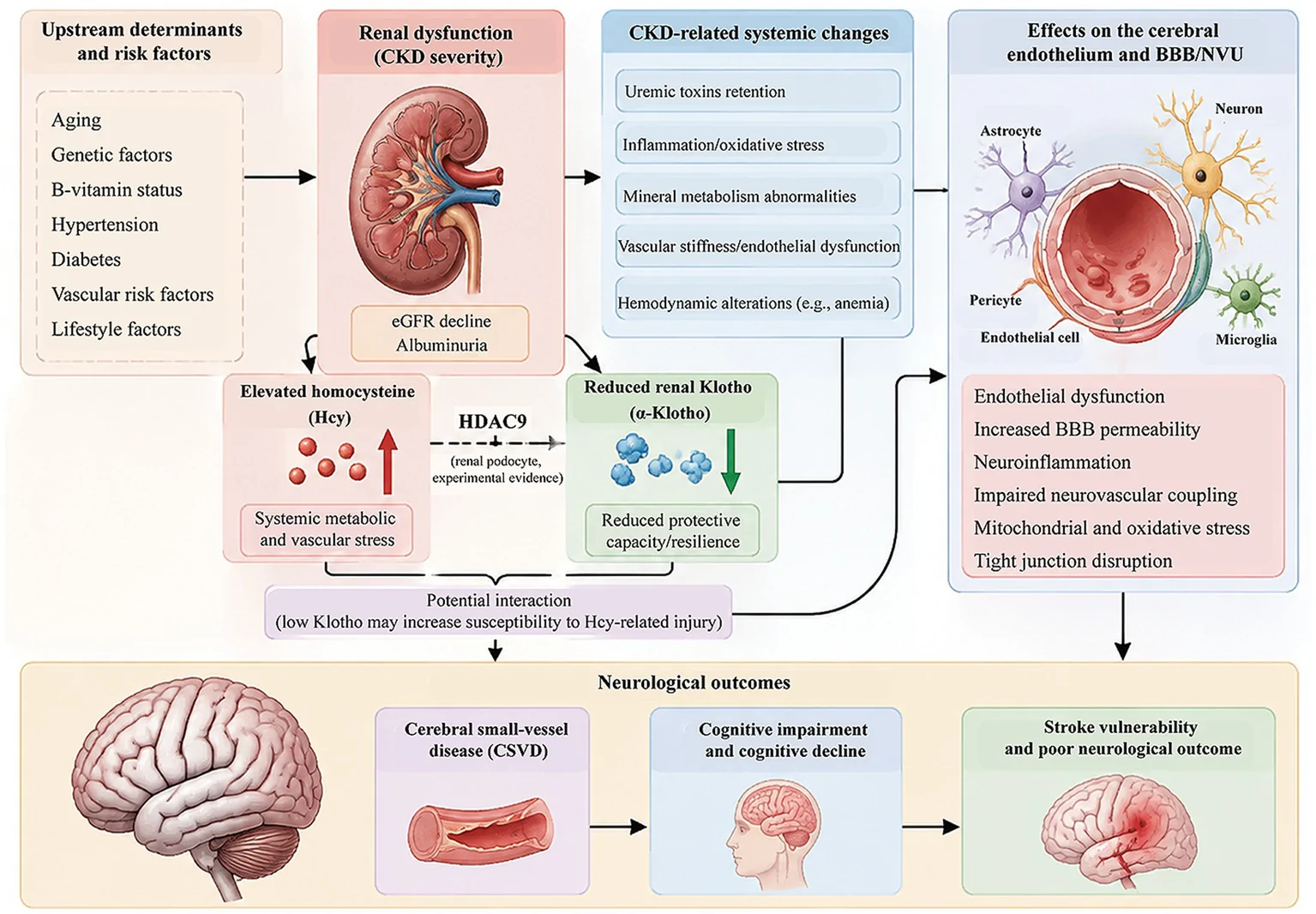
Proposed causal relationships among renal dysfunction, Hcy, Klotho, CKD-related systemic factors, BBB/NVU injury, and neurological outcomes. This diagram illustrates the conceptual structure of the kidney-to-brain stress–resilience framework. Declining renal function, represented by reduced eGFR and albuminuria, is considered an upstream determinant of elevated homocysteine (Hcy), reduced renal Klotho expression, and other CKD-related systemic abnormalities. These abnormalities include uremic toxin accumulation, inflammation, oxidative stress, mineral metabolism disturbance, vascular stiffness, and hemodynamic alterations. Together, these factors may influence the cerebral endothelium and blood–brain barrier/neurovascular unit (BBB/NVU), contributing to cerebral small-vessel disease, cognitive impairment, and stroke vulnerability. Within this framework, Hcy represents a candidate marker of systemic metabolic and vascular stress, whereas Klotho-related measures represent a candidate marker of renal-linked protective capacity or resilience. The HHcy–HDAC9–KL pathway is shown as renal-specific experimental evidence from podocytes and should not be interpreted as an established HHcy–Klotho pathway in brain tissues. The interaction between Hcy burden and Klotho-related resilience remains a hypothesis requiring direct investigation. This model is intended to clarify potential causal relationships and research priorities rather than to establish confirmed biological independence from CKD severity.

Clinical studies should therefore characterize Hcy and Klotho in the context of renal function and other major determinants of both measures. Analyses should account explicitly for eGFR, albuminuria, B-vitamin status, age, blood pressure, inflammation, nutritional status, and vascular disease. Longitudinal designs are preferable because they can establish whether changes in Hcy or Klotho-related measures precede changes in BBB/NVU function, CSVD burden, or cognition. An incremental gain in statistical prediction would be useful, but mechanistic support would be considerably stronger if temporal ordering, biologically plausible effect modification, or mediation through neurovascular intermediate phenotypes could also be demonstrated.

### 6.2 From Hcy lowering to mechanism-based intervention

Hcy is readily modified through folate and related B-vitamin pathways, yet lowering plasma Hcy has not produced uniform neurological benefit. Meta-analyses suggest modest or context-dependent effects on cognition and stroke prevention, with substantial variation according to population, disease stage, baseline Hcy, nutritional background, and vascular risk (Lee et al., 2024; Yang et al., 2024; Berg et al., 2025). The mixed findings from Hcy-lowering studies do not rule out a biological role for Hcy. Reducing circulating Hcy corrects one metabolic abnormality, but it does not necessarily reverse tissue injury that has accumulated over time. Once endothelial dysfunction, BBB injury, CSVD, chronic inflammation, cellular senescence, or neuronal damage is established, lowering Hcy alone may have only a limited effect on neurovascular function.

The potential role of Hcy in neurovascular injury should be interpreted according to the strength of available evidence. Observational studies have linked higher Hcy levels with vascular disease, cerebral small-vessel disease, cognitive impairment, and stroke-related outcomes. However, these associations may be partly explained by renal dysfunction, aging, nutritional status, inflammation, and other vascular risk factors. Therefore, observational studies alone cannot determine whether Hcy directly promotes neurovascular injury or mainly reflects an underlying metabolic and renal disorder.

Genetic studies, including Mendelian randomization analyses, provide additional information about possible causal relationships. However, their interpretation depends on the genetic variants used, the biological pathways they represent, and the clinical outcomes being examined. Genetic evidence related to Hcy metabolism has supported a possible role of Hcy in vascular biology, but it does not fully capture the acquired HHcy state observed in CKD, where metabolic abnormalities, inflammation, and vascular dysfunction occur together.

Randomized trials of Hcy-lowering interventions provide important clinical evidence, but their effects on cardiovascular and neurological outcomes have been modest or inconsistent. These findings suggest that Hcy may contribute to neurovascular stress, while also indicating that Hcy alone is unlikely to explain disease progression. Similarly, evidence linking Klotho-related measures with cognitive aging, vascular disease, and neurological outcomes is mainly observational. Although experimental studies suggest that Klotho may support antioxidant defense, mitochondrial function, and endothelial homeostasis, it remains uncertain whether reduced Klotho is a direct cause of neurovascular dysfunction or a marker of preserved kidney function and healthier aging. Therefore, the HHcy–Klotho framework should currently be viewed as a hypothesis-driven model rather than an established causal pathway. Future studies combining genetic approaches, standardized Klotho measurements, longitudinal follow-up, and intervention studies are needed to determine whether Hcy and Klotho are therapeutic targets or mainly indicators of underlying disease processes.

This limitation is particularly relevant in CKD. Hcy-lowering therapy may improve part of the metabolic disturbance, while other abnormalities persist. Uremic toxin retention, mineral imbalance, vascular calcification and stiffness, oxidative stress, chronic inflammation, and Klotho deficiency may all remain. A recent meta-analysis of randomized trials in patients with CKD found little evidence that Hcy-lowering therapy substantially improves major cardiovascular outcomes or mortality (Lin et al., 2026). The limited benefit may therefore reflect the persistence of a broader abnormal vascular environment rather than Hcy alone. However, current evidence does not show that Klotho deficiency explains this limited response. No clinical trial has yet established whether Klotho status influences the neurological effects of Hcy-lowering treatment.

This possibility has important implications for treatment. Patients with similar Hcy levels may still differ in Klotho status and in the severity of endothelial dysfunction, BBB injury, or CSVD progression. If Klotho-related protection modifies the effects of Hcy, these patients may also respond differently to Hcy-lowering therapy. The opposite may also be possible. Hcy lowering may provide greater neurovascular benefit when the body’s own repair mechanisms are still relatively preserved. These possibilities need to be tested in future studies. At present, there is not enough evidence to support combined Hcy-lowering and Klotho-targeted treatment.

Klotho-targeted treatment is still at an early stage. Protecting kidney function and improving lifestyle may help maintain the body’s own Klotho levels while also improving general health. However, any benefit from these measures cannot be linked specifically to Klotho. More direct methods include drugs that increase Klotho production, recombinant Klotho, Klotho-derived peptides, and gene-based treatments. Most of these approaches are still being tested in experimental or preclinical studies (Poursistany et al., 2024). Their use in neurological disease is also difficult because Klotho in different tissues and body fluids is not the same. Increasing Klotho in the kidney or blood may not restore Klotho activity in cerebral endothelial cells, the choroid plexus, or neural tissue.

At present, it may be more useful to group patients according to their biological features than to use Hcy-lowering and Klotho-targeted treatments together without clear evidence. Patients could be assessed based on Hcy levels, carefully measured Klotho status, kidney function, nutritional and inflammatory status, vascular risk, and signs of cerebral endothelial or small-vessel injury. Intervention studies could then examine whether changes in these blood markers occur together with changes in BBB permeability, cerebral perfusion, neurovascular coupling, CSVD burden, or cognition. This approach may help determine whether Hcy and Klotho are only associated with disease or actually affect the kidney-to-brain process.

At present, the HHcy-Klotho model is more useful as a way to frame the problem than as a guide to treatment. It proposes that neurological vulnerability depends not only on the level of systemic metabolic stress, but also on the ability of vascular and neural tissues to tolerate and recover from that stress. Considering both factors together may provide more information than examining each metabolic abnormality separately. Future studies should test whether this balance helps explain differences in neurovascular injury beyond kidney function and established vascular risk factors. Another important question is whether the model can identify an early stage at which BBB/NVU dysfunction, CSVD progression, or cognitive decline can still be slowed or prevented.

## 7 Research priorities for testing the kidney-to-brain HHcy-Klotho stress-resilience framework

Studies have linked renal dysfunction, HHcy, and Klotho with neurological outcomes, but most of the evidence comes from separate associations rather than a single confirmed pathway. The sequence and location of these changes are still uncertain and need to be tested directly in experimental models. Our working hypothesis is that declining renal function increases systemic metabolic stress while weakening Klotho-related protection. The cerebral endothelium, BBB, and NVU are possible sites where these effects may influence the brain. Associations of Hcy and Klotho with neurological disease alone cannot establish this model. What remains to be determined is whether the two factors interact, where such an interaction occurs, whether it appears before measurable neurovascular injury, and whether altering either factor changes disease progression (Table 5).

**Table 5:** Research priorities translated into decisive experimental and longitudinal tests.

Research priority	Primary question	Recommended model/Design	Key manipulation or exposure	Primary readouts	Result that would most strongly support the framework
Brain-facing HHcy→Klotho regulation	Does Hcy directly regulate KL/Klotho in cerebral endothelial or other brain-interface cells?	Human/rodent brain microvascular endothelial cells; choroid-plexus epithelial cells; pericytes; astrocytes; microglia; neurons.	Controlled Hcy exposure with independent Klotho gain- and loss-of-function; rescue experiments; renal/inflammatory/nutritional conditions controlled.	KL transcription; promoter methylation; histone acetylation; chromatin accessibility; Klotho protein/shedding; mitochondrial function; inflammatory activation; tight-junction integrity; permeability.	Klotho restoration specifically attenuates Hcy-induced endothelial/BBB injury, with a defined molecular mechanism.
Resolve Klotho compartments	What do serum, urine, renal-tissue, CSF and local CNS Klotho each represent, and how do they relate over time?	Longitudinal human cohorts across kidney-function strata; paired biofluids/tissue where feasible.	Repeated standardized Klotho measurements plus Hcy and one-carbon/sulfur-amino-acid metabolites.	eGFR; albuminuria; B-vitamin status; inflammation; endothelial markers; vascular risk; optional multi-omics.	A compartment-specific Klotho measure reproducibly explains variation in the cerebral response to a defined Hcy burden.
Define the NVU cellular sequence	Which cells sense systemic HHcy first, and how is dysfunction propagated across the NVU?	Multicellular BBB/NVU models; cell-specific KL loss/gain of function; single-cell/spatial profiling.	Time-resolved Hcy and Klotho manipulation.	Endothelial mitochondrial stress; tight-junction changes; pericyte/astrocyte responses; microglial activation; neuronal substrate/signaling changes.	Endothelial changes precede or accompany downstream NVU responses, and Klotho manipulation alters propagation.
Longitudinal neuroimaging bridge	Do systemic Hcy/Klotho changes precede measurable neurovascular deterioration *in vivo*?	Prospective CKD-enriched cohort spanning preserved to advanced renal dysfunction.	Repeated Hcy, compartment-defined Klotho, renal/inflammatory/vascular measurements.	DCE-MRI BBB permeability; ASL perfusion; fMRI neurovascular coupling; WMH, lacunes, microbleeds, EPVS, atrophy; cognition.	Hcy change temporally precedes BBB/NVU deterioration, with Klotho modifying that relationship and intermediate imaging phenotypes mediating later injury.
Causal and interventional testing	Does modifying stress or resilience change the predicted cerebral phenotype?	Prespecified causal models; mechanistic trials rather than immediate empirical dual therapy.	Hcy-lowering intervention stratified by baseline Klotho and/or interventions that preserve or increase Klotho-related activity.	Endothelial activation; BBB permeability; perfusion/NVC; quantitative CSVD progression; cognition.	Intervention effects on mechanistic intermediate phenotypes differ by Klotho status in the direction predicted by the stress–resilience model.

### 7.1 Establish whether HHcy directly regulates Klotho in brain-facing cells

The first step is to determine whether the HHcy-Klotho regulatory relationship found in the kidney also exists in the brain or at brain-related interfaces. In renal podocytes, HHcy suppresses KL through an HDAC9-dependent epigenetic mechanism (Liu et al., 2023). This provides direct molecular evidence of an HHcy-Klotho interaction in the kidney. In brain microvascular endothelial cells, Hcy can also cause epigenetic changes. These changes involve Cacna1c/Cav1.2 signaling and are associated with altered calcium signaling, ROS production, loss of tight-junction proteins, BBB damage, and cognitive impairment (Zhou et al., 2026). Together, these observations establish that HHcy can regulate Klotho epigenetically in a renal cell type and can induce epigenetic injury in cerebral endothelial cells, but they do not establish an HHcy-Klotho regulatory pathway within the BBB or NVU.

A critical experiment is therefore to determine whether Hcy exposure alters KL transcription, promoter methylation, histone acetylation, chromatin accessibility, Klotho protein abundance, or shedding in cerebral endothelial cells, choroid-plexus epithelial cells, pericytes, astrocytes, microglia, and neurons. Hcy and Klotho should be manipulated independently and jointly, with renal, inflammatory, and nutritional conditions experimentally controlled. Rescue experiments are particularly important: if Klotho restoration attenuates Hcy-induced endothelial mitochondrial dysfunction, inflammatory activation, tight-junction loss, or barrier leakage, this would provide substantially stronger evidence for biological interaction than parallel changes in the two molecules alone. Conversely, failure of Klotho manipulation to modify Hcy-induced injury would argue against a direct HHcy-Klotho mechanism at that cellular interface.

### 7.2 Resolve Klotho compartments and quantify the systemic stress-resilience state

An important problem is that Klotho is found in different parts of the body. Serum soluble Klotho, urinary Klotho, renal KL expression, CSF Klotho, and Klotho produced within the CNS do not represent the same biological state. The relationships among these measures may also change with CKD stage, aging, inflammation, or neurological disease. Therefore, Klotho measured in the blood should not be assumed to reflect Klotho levels in kidney tissue or Klotho activity in the brain.

Longitudinal studies in humans should use standardized Klotho measurements together with Hcy and related one-carbon or sulfur-amino-acid metabolites. Kidney function, albuminuria, B-vitamin status, inflammatory and endothelial markers, and common vascular risk factors should also be measured. Multi-omics may provide additional information about the underlying mechanisms when it is used to test clear hypotheses about the order of these changes (Ceballos et al., 2025). For example, metabolomic and epigenomic data could show whether long-term HHcy occurs before changes in KL regulation or endothelial stress responses. Proteomic data could then show whether these changes occur together with inflammatory, vascular, or senescence-related responses.

The possible interaction between Hcy and Klotho should be defined before the analysis rather than examined only after separate biomarker associations are found. The main prediction is that the same Hcy level may be associated with different degrees of BBB leakage, CSVD progression, or cognitive decline at different Klotho levels. Hcy and Klotho should preferably be analyzed as continuous variables instead of being divided too early into high and low groups. The KL-VS genotype may add useful information, but it does not replace direct measurement of Klotho protein. Genotype, circulating soluble Klotho, and tissue expression reflect different levels of Klotho biology (Driscoll et al., 2024; Liu et al., 2025). A universal HHcy-Klotho ratio is therefore unlikely to capture this complexity. The more relevant question is whether Klotho-related measures can explain why similar systemic Hcy exposure is associated with different cerebral responses.

### 7.3 Identify the cellular sequence that translates systemic dysfunction into NVU injury

Another important question is the order in which different NVU cells respond. Single-cell and spatial studies in CKD kidneys have shown clear differences in cell states and inflammatory communication between cells (Fan et al., 2024; Reck et al., 2025). These kidney studies do not prove that the same process occurs in the brain, but they provide a useful research model. Similar studies could be carried out in cerebral endothelial cells, pericytes, astrocytes, microglia, and other NVU cells. Single-cell, spatial-transcriptomic, proteomic, and epigenomic methods could be used under controlled Hcy and Klotho conditions. These studies may help identify which cells respond first and how the response spreads to other cells.

The timing of these changes is also important. If cerebral endothelial cells are the first cells to respond to signals in the blood, mitochondrial stress, inflammatory activation, or tight-junction damage should appear before, or at the same time as, later changes in pericytes, astrocytes, microglia, and neurons. Multicellular BBB and NVU models could test whether changes in endothelial cells are enough to affect nearby cells. They could also test whether changes in Klotho activity alter this process. Cell-specific KL loss- and gain-of-function experiments, together with controlled Hcy exposure, could further clarify the roles of endothelial cells, peripheral blood vessels, glial cells, and neurons. A similar Hcy exposure may not produce the same response in endothelial cells, glial cells, and neurons. Cell-specific KL loss- and gain-of-function experiments could help identify where Klotho provides its main protective effect. This protection may occur at the cerebral endothelium, in neural or glial cells, through peripheral vascular pathways, or at more than one site.

### 7.4 Use longitudinal neuroimaging to bridge peripheral biology and cerebral outcomes

Neuroimaging offers a practical way to examine how peripheral abnormalities are related to changes in the brain. BBB permeability can be quantified with dynamic-contrast-enhanced MRI, whereas arterial-spin-labeling and functional MRI provide information on cerebral perfusion and neurovascular coupling. Structural MRI adds measures of white-matter hyperintensities, lacunes, microbleeds, enlarged perivascular spaces, brain atrophy, and overall CSVD burden (Kern et al., 2024; Li et al., 2024; Liu et al., 2024a; Verstappen et al., 2025). Together, these measures allow neurovascular changes to be followed before dementia or stroke becomes clinically evident. A prospective cohort could collect these imaging measures together with Hcy, Klotho from defined biological compartments, kidney function, inflammatory and vascular markers, and cognitive assessments at several time points in the same participants. Such follow-up would help establish whether an increase in systemic Hcy comes before worsening neurovascular function and whether this association changes with Klotho status. It could also show whether abnormalities in the BBB or NVU appear before later structural brain injury or cognitive decline. CKD cohorts are particularly useful for this type of study because renal function varies widely among patients and can change considerably during follow-up. Recruitment should therefore include patients at different stages of kidney dysfunction rather than focusing only on end-stage disease. Studying this broader range may help identify when the proposed stress-resilience imbalance first becomes detectable. It may also show whether systemic metabolic changes appear before cerebral microvascular injury becomes persistent or irreversible.

### 7.5 Move from statistical interaction to causal and interventional testing

Finally, causal modeling should be prespecified around the biological question rather than relying on routine covariate adjustment. Renal function is particularly challenging because, depending on the causal question, it may act as an upstream determinant of Hcy and Klotho, a marker of the broader CKD-associated systemic milieu, a source of confounding, or an effect modifier of their neurological associations. Its role should therefore be defined using an explicit causal structure and estimand rather than simply “adjusted away” in all analyses.

The strongest human test of the proposed framework is not whether Hcy and Klotho independently predict neurological disease, but whether prespecified Hcy and Klotho effect modification explains variation in BBB integrity, neurovascular coupling, CSVD progression, or cognitive decline beyond renal function and conventional vascular risk factors. Statistical interaction alone, however, would not establish a molecular HHcy-Klotho interaction. Stronger causal inference would require convergence among temporal ordering in longitudinal cohorts, compartment-specific biomarker relationships, mechanistic intermediate phenotypes, and experimental perturbation.

Intervention can provide a stronger test once the relevant biological relationships have been defined. Early trials do not need to begin with empirical combination therapy. A more informative design would test whether reducing Hcy produces different changes in BBB/NVU phenotypes according to baseline Klotho status, or whether an intervention that preserves or increases Klotho-related activity alters the neurovascular response to HHcy. Endothelial activation, BBB permeability, cerebral perfusion, neurovascular coupling, and quantitative CSVD progression should be included as intermediate outcomes rather than relying only on late clinical events.

No single experiment is likely to validate the framework. Support would need to converge across levels of evidence: cellular studies should identify whether and where Hcy and Klotho interact; compartment-specific measurements should clarify what circulating and tissue Klotho represent; longitudinal imaging should establish the temporal relationship between systemic changes and neurovascular deterioration; and causal or interventional studies should determine whether modifying the proposed stress-resilience balance changes the cerebral phenotype. Until evidence at these levels is available, HHcy-Klotho is better regarded as a testable kidney-to-brain framework than as an established molecular axis.

## 8 Limitations of the current framework

Several limitations should be acknowledged when considering the proposed stress–resilience framework. Most available human studies examining the relationships among HHcy, Klotho, kidney dysfunction, and neurological outcomes are observational, and whether these factors are causally linked remains uncertain. In addition, Klotho measurements from different biological compartments may reflect distinct aspects of Klotho biology, and circulating Klotho levels alone may not accurately represent renal or CNS Klotho function. CKD is also associated with a range of metabolic, inflammatory, vascular, and hemodynamic changes that may independently affect neurovascular health and influence the observed associations between HHcy, Klotho, and brain outcomes. Moreover, direct evidence showing that Klotho modifies Hcy-related neurovascular injury in humans is currently unavailable. Therefore, this framework should be viewed as a conceptual model that requires further evaluation through mechanistic studies and longitudinal clinical studies combining renal assessment, molecular biomarkers, neurovascular imaging, and cognitive outcomes.

## 9 Conclusion

HHcy and Klotho can be integrated within a kidney-to-brain stress-resilience framework in which renal dysfunction simultaneously shapes systemic metabolic stress and the capacity to maintain vascular and neural homeostasis. Declining renal function favors Hcy accumulation and reduced renal Klotho expression, while circulating Hcy can directly impair cerebral endothelial and BBB function. Klotho-related mechanisms, in contrast, are associated with antioxidant, mitochondrial, endothelial, anti-inflammatory, and anti-senescent resilience, although the contribution of circulating Klotho to human brain disease remains incompletely defined and should be distinguished from local CNS Klotho. Together, these observations support the cerebral endothelium-BBB-NVU interface as a plausible site at which renal dysfunction-associated systemic disturbances can be translated into neurological vulnerability. Clinical evidence from CKD, CSVD, cognitive impairment, and ischemic stroke supports individual components of this proposed continuum, but not yet the complete causal mechanism.

The key unresolved issue is whether the relationship between HHcy-associated stress and Klotho-related resilience is more than parallel association. It is not yet known whether Hcy directly regulates KL in cerebral endothelial or other NVU cells, whether Klotho changes the neurovascular response to a defined Hcy exposure, or whether individuals with higher Hcy and lower Klotho show faster longitudinal deterioration in BBB/NVU function, CSVD burden, or cognition after renal function and conventional vascular risk factors are taken into account. For now, the combined HHcy-high/Klotho-low state should be treated as a candidate metabolic stress-resilience phenotype rather than an established molecular axis. Cell-specific perturbation, compartment-resolved Klotho measurement, longitudinal neuroimaging, and prospective analyses of Hcy-by-Klotho effect modification will be required to determine whether the proposed relationship is causal.

Viewed in this way, kidney-to-brain communication is not reduced to the behavior of a single circulating biomarker. The neurological effect of renal and metabolic dysfunction may depend both on the intensity of systemic injury and on the ability of vascular and neural tissues to maintain and restore homeostasis. Testing these two dimensions across the kidney-circulation-BBB/NVU-brain continuum may help explain why apparently similar peripheral metabolic disturbances are associated with different neurovascular outcomes. It may also identify stages at which progression toward CSVD and cognitive decline remains amenable to intervention.
